# Asymmetrical Dimethylarginine - More Sensitive than NT-proBNP to Diagnose Heart Failure in Adults with Congenital Heart Disease

**DOI:** 10.1371/journal.pone.0033795

**Published:** 2012-03-21

**Authors:** Oktay Tutarel, Agnieszka Denecke, Stefanie M. Bode-Böger, Jens Martens-Lobenhoffer, Svjetlana Lovric, Johann Bauersachs, Bernhard Schieffer, Mechthild Westhoff-Bleck, Jan T. Kielstein

**Affiliations:** 1 Department of Cardiology and Angiology, Hannover Medical School, Hannover, Germany; 2 Department of Nephrology and Hypertension, Hannover Medical School, Hannover, Germany; 3 Institute for Clinical Pharmacology, Otto-von-Guericke University, Magdeburg, Germany; I2MC INSERM UMR U1048, France

## Abstract

**Background:**

Chronic heart failure is an important cause for morbidity and mortality in adults with congenital heart disease (ACHD). While NT-proBNP is an established biomarker for heart failure of non-congenital origin, its value in ACHD has limitations. Asymmetrical dimethylarginine (ADMA) correlates with disease severity and independently predicts adverse clinical events in heart failure of non-congenital origin. Its role in ACHD has not been investigated.

**Methods:**

In 102 patients ADMA and NT-proBNP were measured and related to NYHA class, systemic ventricular function and parameters of cardiopulmonary exercise testing.

**Results:**

In contrast to NT-proBNP ADMA differentiated between NYHA classes I-III. Both, ADMA and NT-proBNP showed a good correlation with parameters of cardiopulmonary exercise testing with comparable receiver-operating characteristic curves for identifying patients with severely limited cardiopulmonary exercise capacity.

**Conclusion:**

ADMA seems to be a better biomarker than NT-proBNP for the assessment of NYHA class and as a good as NT-proBNP for the estimation of maximum exercise capacity in adults with congenital heart disease. Its use in clinical routine should be evaluated.

## Introduction

Chronic heart failure is an important cause for morbidity and mortality in adults with congenital heart disease (ACHD) [1]. However, heart failure symptoms especially in the early stages of the disease do not correlate with objective measures like systemic ventricular function or parameters of cardiopulmonary exercise testing [2], [3]. The rather rare incidence of individual malformations, the abnormal anatomy and the complex physiology make assessment of cardiac function difficult [4]. Therefore, the prevalence of heart failure in these patients is underappreciated [5], [6]. A simple investigation such as a blood test to quantitatively or qualitatively evaluate subclinical/early stages of heart failure or predict those at risk of deterioration would be clinically valuable [4]. B-type natriuretic peptide (BNP) and N-terminal pro-BNP (NT-proBNP) are established tools for diagnosis and management of heart failure due to acquired heart disease [7]. Unfortunately, the clinical use of those markers is hampered in adults with congenital heart disease by several limitations. Hence the diagnosis and treatment monitoring is frequently based on cardiopulmonary exercise testing [4] [5], [8], [9]. Asymmetrical dimethylarginine (ADMA) has emerged as a new biomarker in heart failure of non-congenital origin. Observations in laboratory animals suggested that plasma ADMA levels are increased in experimental heart failure [10]. The association between high ADMA levels and heart failure in humans has been first described almost ten years ago [11]. Recent studies proved, that ADMA correlates with disease severity and independently predicts adverse clinical events in heart failure [12]–[14]. Further, ADMA correlates with parameters of impaired exercise capacity and reduced ventilatory capacity [13]. Its role in ACHD has not been studied. Therefore, the aim of this cross-sectional study in adults with congenital heart disease was to elucidate the potential diagnostic value of ADMA.

## Materials and Methods

The patients were recruited during a routine outpatient visit at the Adult Congenital Heart Disease Clinic of the Hannover Medical School. All patients in whom a venous blood sampling was feasible were eligible for this study. The study was approved by the local Ethics Committee of Hannover Medical School, Germany. All patients gave written informed consent.

A clinical workup including medical history, physical examination, 12-lead electrocardiography, transthoracic echocardiography and cardiopulmonary exercise testing was performed.

The severity of the congenital heart defect was graded according to complexity as proposed by recent guidelines [15]. The patients were further classified according to their symptoms of heart failure using the New York Heart Association (NYHA) functional classification. The NYHA classification is based on the symptoms of the patients and the limitations to normal physical activities [7].

### Laboratory methods

Blood samples for measurement of plasma ADMA and NT-proBNP, and routine chemistry were drawn. Blood samples were immediately cooled on ice, centrifuged at 1,500 g and 4°C for 10 min. Supernatants were stored in 1 ml aliquots at −80°C until further use.

Plasma concentrations of ADMA were measured applying a liquid chromatography-mass spectrometry method described elsewhere [16]. The lower limit of quantification for ADMA was 0.15 µmol/l and the inter-batch precision and accuracy was better than 6%.

All other measurements were done with routine laboratory tests using certified assay methods.

### Echocardiography

A standard 2D-Doppler transthoracic echocardiogram was performed according to the recommendations for the assessment of ventricular function and valvular heart disease issued by the American Society of Echocardiography [17]. Systemic ventricular systolic function was assessed qualitatively (i.e. normal, moderately or severely impaired). Valvular function was quantified by color and continuous-wave Doppler flow.

### Cardiopulmonary exercise studies

Cardiopulmonary exercise studies were performed on a bicycle in sitting position, starting with 25 W, increasing further 25 W every 2 min. All patients exercised to the end of their tolerance. A 12-lead ECG was recorded throughout the exercise test to determine heart rate and increase in heart rate. Systolic blood pressure and increase in systolic blood pressure, as well as work rate (W/kg) were measured. Ventilation, oxygen uptake (VO_2_), and carbon dioxide production (VCO_2_), were measured continuously by a breath-by-breath method. Subjects breathed through a fitted mask and a hot-wire anemometer (Oxycon Delta, Jäger, Hoechberg, Germany) measuring inspired and expired flow continuously.

### Statistical analysis

We used SPSS 15.0 and R for statistical analysis. Continuous data are presented as mean ± standard deviation. Categorical data are presented as counts and proportions. Patient demographic and clinical characteristics were summarized as means ± standard deviation. Comparisons between groups were done using unpaired Student's *t* test for continuous and Mann-Whitney-U test for categorical variables. If more than two groups were compared one-way ANOVA or Kruskal-Wallis-test were used depending on the distribution of the data. For correlation Pearson's correlation coefficient was calculated. The significance level was set at p<0.05 and was two-sided.

For the parameters of cardiopulmonary exercise testing cut off values representing patients with limitations of their cardiopulmonary exercise capacity were defined: peak oxygen uptake (peak VO_2_) <20 ml/min/kg, ventilatory equivalent for carbon dioxide (EQCO_2_)>34, ventilatory equivalent for oxygen (EQO_2_)>34, oxygen pulse for female <9 ml/heartbeat, for male <12 ml/heartbeat. For further analysis a group of patients that was severely affected was defined. These patients had a peakVO_2_<20 ml/min/kg or an EQCO_2_>34 or a combination of both. Univariate logistic regression analysis was performed. Parameters with a p value<0.1 were included into a multivariate logistic regression analysis. Receiver-operating characteristic curves (ROC curve) for these parameters were drawn and the areas under the curves calculated.

## Results

One hundred and two patients were enrolled in our cross-sectional study. Due to incomplete data sets 5 patients were excluded. Because of the small number (n = 3) of patients with NYHA IV these were also excluded. Hence, the final analysis is based on 94 patients. **Table 1** **and** **2** show the clinical characteristics of the study population. Cardiopulmonary exercise testing was performed in 72 patients.

**Table 1:** Clinical characteristics of study population.

Age (yrs)	30.2±10.6
BMI (kg/m^2^)	23.5±4.2
*Sex*	
female	39 (41.5)
male	55 (58.5)
*Complexity of congenital heart disease*	
simple	19 (20.2)
moderate	36 (38.3)
severe	39 (41.5)
*Systemic ventricle*	
left	67 (71.3)
right	12 (12.8)
single ventricle	15 (16)
*Systemic ventricular function*	
normal	52 (55.3)
moderately impaired	35 (37.2)
severely impaired	7 (7.4)
*NYHA class*	
NYHA I	56 (59.6)
NYHA II	21 (22.3)
NYHA III	17 (18.1)

Data are expressed as mean±SD or as counts (percentage).

**Table 2:** Type of congenital heart defect.

Congenital heart defect	Number (%)
TGA after Mustard and CCTGA	11 (11.7)
Tetralogy of Fallot	12 (12.8)
Coarctation of the aorta	11 (11.7)
Atrial or ventricular septal defect	10 (10.6)
Atrioventricular septal defect	6 (6.4)
Marfan syndrome	8 (8.5)
Congenital aortic or pulmonary valve stenosis	13 (13.8)
Single ventricle physiology	13 (13.8)
Miscellaneous	10 (10.6)

TGA = transposition of the great arteries; CCTGA = congenital corrected transposition of the great arteries; miscellaneous: Ebstein's anomaly, subaortic stenosis, pulmonary atresia.

### Clinical characteristics possibly influencing ADMA and NT-proBNP levels

Renal function assessed by the Chronic Kidney Disease Epidemiology Collaboration (CKD-EPI) equation [18] did not differ between patients in NYHA I, NYHA II and NYHA III (**Table 3**). Arterial hypertension was equally prevalent in all three groups. Only 1 patient was diabetic.

**Table 3:** Clinical characteristics according to NYHA class.

	NYHA I	NYHA II	NYHA III	p
GFR ml/min	116±14	116±11	107±18	n.s.
arterial hypertension No. (%)	7 (14%)	2 (11%)	3 (21%)	n.s.

GFR = glomerular filtration rate calculated with the Chronic Kidney Disease Epidemiology Collaboration (CKD-EPI) equation; n.s. = non-significant.

### Ventricular function

ADMA did not reach a statistically significant difference in patients with severe ventricular dysfunction (0.50±0.14 µmol/l) compared to patients with moderate dysfunction (0.47±0.08 µmol/l, p = 0.36) and normal ventricular function (0.46±0.07 µmol/l, p = 0.23). NT-proBNP was elevated in severe ventricular dysfunction compared to moderately impaired ventricular function (1156±1540 pg/ml vs. 379±466 pg/ml, p<0.001) and normal ventricular function (142±177 pg/ml, p<0.001) and also between the later two (p = 0.037).

### NYHA class

**Figure 1:**
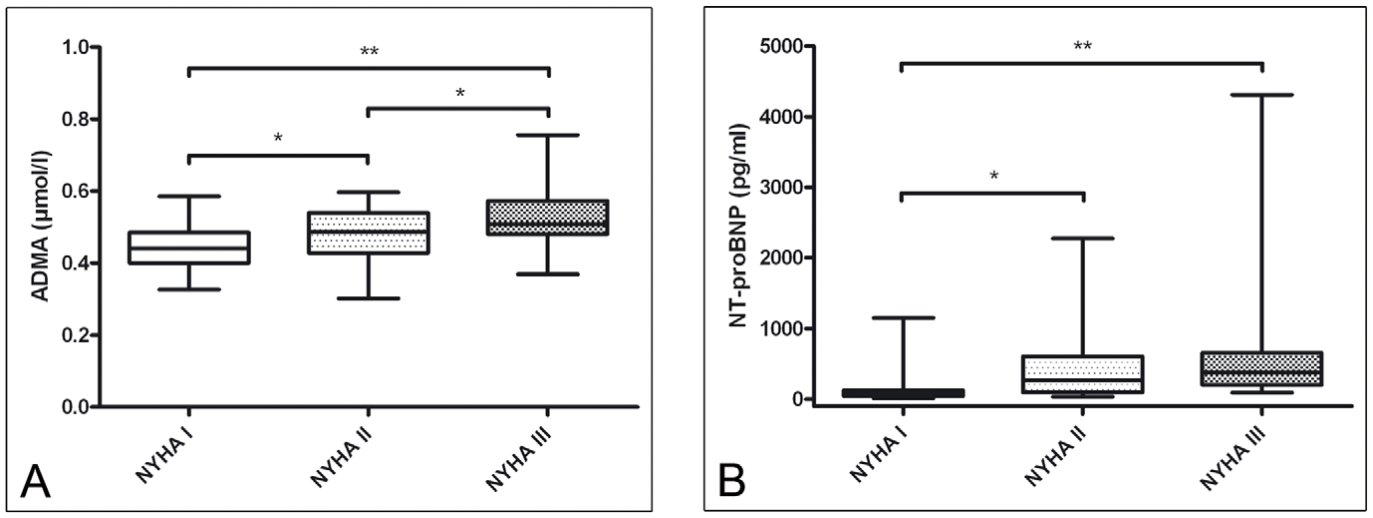
ADMA and NT-proBNP in comparison between NYHA classes. (* p<0.05, ** p<0.001)

ADMA differentiated between NYHA classes: NYHA I (0.44±0.06 µmol/l) to NYHA II (0.48±0.08 µmol/l, p = 0.04), and NYHA III (0.54±0.10 µmol/l, p<0.001) and between NYHA II and III (p = 0.02). (**Figure 1**) NT-proBNP was significantly lower in patients with NYHA I (129±202 pg/ml) compared to patients with NYHA II (432±517 pg/ml, p = 0.026) and NYHA III (719±1035 pg/ml, p<0.001), but not between the later two (p = 0.97). (**Figure 1**)

### Cardiopulmonary exercise testing

Peak VO_2_ was significantly higher in patients in NYHA I (28.8±7.6 ml/min/kg) vs. patients in NYHA II (23.3±4.5 ml/min/kg, p = 0.007) and patients in NYHA III (14.1±5.2 ml/min/kg, p<0.001) and also in comparison between NYHA II and NYHA III (p = 0.001). When grouped according to ventricular function patients with a normal function had a significantly higher peak VO_2_ (28.5±7.6 ml/min/kg) compared to patients with a moderate (21.5±8.2 ml/min/kg, p = 0.001) or severe impairment of their ventricular function (21.0±8.4 ml/min/kg, p = 0.023). There was no significant difference between the later two (p = 0.879).

ADMA was elevated in patients with limited cardiopulmonary exercise capacity compared to their peers. Significant differences were observed for peak VO_2_ (p = 0.004), EQCO_2_ (p = 0.002) and EQO_2_ (p = 0.005). There was not a statistically significant difference for oxygen pulse (p = 0.088) (**Table 4**).

**Table 4:** ADMA and NT-proBNP in patients with limitations of their cardiopulmonary exercise capacity.

	ADMA in µmol/l			NT-proBNP in pg/ml		
	*<cut off*	*> cut off*	*p*	*<cut off*	*> cut off*	*p*
**peak VO_2_ in ml/min/kg**	0.50±0.08	0.44±0.06	0.004	644±999	203±371	<0.001
**EQCO2**	0.45±0.06	0.53±0.09	0.002	232±408	868±1258	0.005
**EQO2**	0.44±0.06	0.50±0.08	0.005	187±312	620±979	0.005
**Oxygen pulse in ml/beat**	0.48±0.10	0.45±0.05	0.088	601±927	151±209	<0.001

**Figure 2:**
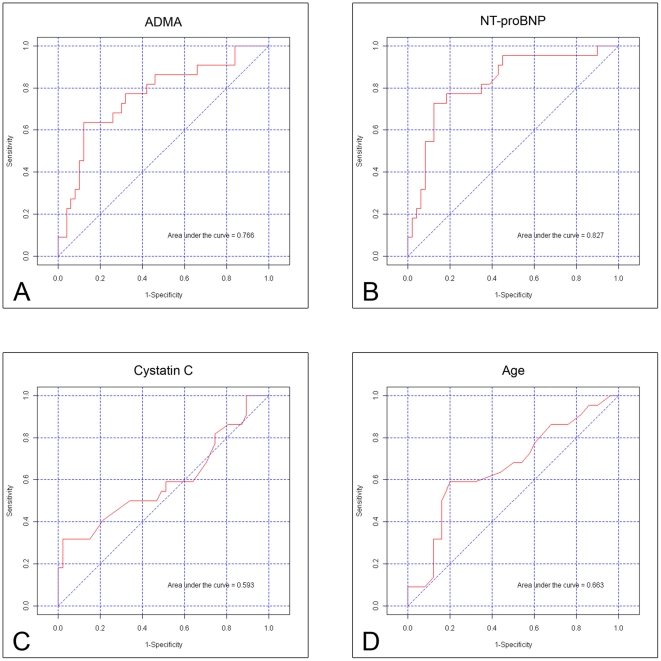
Receiver-operating characteristic (ROC) curves for identifying patients with severely limited cardiopulmonary exercise capacity.

Uni-variate logistic regression analysis regarding the ability to identify patients with severely limited cardiopulmonary exercise capacity (defined as peakVO_2_<20 ml/min/kg or an EQCO_2_>34 or a combination of both) revealed four parameters with a p value<0.1: age, ADMA, cystatin C and NT-proBNP (**Table 5**). The areas under the receiver-operating characteristic (ROC) curves for identifying patients with severely limited cardiopulmonary exercise capacity were 0.663 for age, 0.593 for cystatin C, 0.766 for ADMA, and 0.827 for NT-proBNP (**Figure 2**). Optimal cut off values for ADMA and NT-proBNP were 0.502 µmol/l and 250 pg/ml respectively. Multivariate logistic regression analysis revealed that only ADMA (p logreg = 0.0081) and NT-proBNP (p logreg = 0.0087) showed a significant influence.

**Table 5:** Results of univariate logistic regression analysis for different parameters regarding their ability to identify patients with severely limited cardiopulmonary exercise capacity.

	Odds ratio	95% confidence intevall		p logreg
**age**	1.0678	1.0069	1.1324	0.0285
**body mass index**	0.9025	0.7874	1.0345	0.1409
**AST**	0.9694	0.9159	1.026	0.2826
**cystatin C**	269.5165	0.8392	86552.8027	0.0574
**NT-proBNP (log.)**	2.8467	1.6394	4.9432	0.0002
**ADMA**	5533225.887	368.0284	83190842073	0.0016
**sex**	0.4286	0.1528	1.2024	0.1075

AST = aspartat aminotransferase.

## Discussion

In this study, ADMA was for the first time evaluated in adults with congenital heart disease. It was significantly elevated in ACHD that bear the hallmarks of heart failure. This holds true for subjective measures of heart failure like NYHA class as well as objective measures like cardiopulmonary exercise parameters. Especially, ADMA was superior to NT-proBNP in differentiating NYHA classes.

In patients with chronic heart failure of non-congenital origin a correlation of elevated ADMA concentrations with impaired exercise capacity was recently reported. In a study of 113 patients elevated ADMA concentrations were associated with lower peak VO_2_, increased VE/VCO_2_ slope, and shorter exercise duration on the treadmill [13]. This is in accordance with our results that ADMA was elevated in ACHD with limited cardiopulmonary exercise capacity compared to their peers. ADMA further was able to distinguish patients with an especially impaired exercise capacity demonstrated by lower peak VO_2_ and increased EQCO_2_. The area under the receiver-operating characteristic curve is 0.766. The importance of this finding is demonstrated by the fact that poor exercise capacity identifies ACHD at risk for hospitalization or death [2]. Peak VO_2_ predicted hospitalization or death and was related to the frequency and duration of hospitalization in a large cohort of ACHD [2]. Further, an increased ventilatory response to exercise is also a powerful predictor of mortality in ACHD [19]. NT-proBNP displayed also a significant difference between patients with a peak VO_2_ or EQCO_2_ under or over the cut off value. This is in contrast to the findings of Larsson et al. that the ability of elevated BNP or NT-proBNP levels in ACHD with a systemic right ventricle or a single ventricle to identify those with impaired exercise capacity was weak [4].

In our study, ADMA increased in correlation with NYHA class. Usui et al. demonstrated that ADMA correlated with NYHA class in chronic heart failure of non-congenital origin [20]. Further, Norozi et al. showed that there is an incremental risk to exhibit heart failure with rising NYHA class in ACHD [3]. The odds ratio for patients in NYHA II compared to patients in NYHA I was 3.4 and for patients in NYHA III 11.6 [3]. This provides us with further evidence that ADMA can act as a surrogate marker for heart failure in ACHD. In contrast, NT-proBNP was not able to differentiate between the NYHA classes in our study.

Regarding the association between systemic ventricular function and ADMA levels an increase of ADMA levels in parallel with worsening ventricular function was observed. This was however not statistical significant. A larger sample size would probably lead to statistically significant results. Moreover, echocardiographic assessment of ventricular function is difficult in these patients [4]. In our study there was a good correlation between ventricular function and NT-proBNP concentrations. This is in contrast to the finding of Larsson and colleagues [4]. In their study, subjects with moderately or severely impaired ventricular function did have elevated BNP/NT-proBNP concentrations as compared with subjects with normal or only mildly impaired ventricular function, but the association was weak and only statistically significant when BNP and NT-proBNP data were combined [4]. It appeared that BNP/NT-proBNP had especially poor discrimination in evaluating differences between patients with no or mild ventricular impairment [4], which suggests a limited ability of BNP/NT-proBNP to diagnose heart failure at the initial stages.

Although the very nature of this clinical analysis is prohibitive for making pathophysiological assumptions, several published papers pointed to potential links between ADMA and heart failure. ADMA is formed when protein-incorporated arginine is methylated by the enzymes protein arginine methyltransferases (PRMT) [12]. Nitric oxide is formed from the amino acid arginine by the enzyme nitric oxide synthase (NOS) [12]. ADMA is the most potent endogenous nitric oxide synthase (NOS) inhibitor [21], [22] and acts by competing with arginine for NOS binding [12].

Nitric oxide (NO) is involved in the modulation of all regulatory steps of excitation-contraction coupling in the heart [23] and leads to cGMP-mediated relaxation and vasodilation [12]. Elevated ADMA levels have been found in a variety of cardiac diseases [12]. Further, systemic ADMA infusions lead to a decrease in cardiac output in healthy volunteers [24]. Furthermore, ADMA infusion has been shown to impair relaxation of coronary arteries, induce myocardial remodelling, deteriorate cardiac function, and cause myocardial ischaemia [12]. Endogenous NO synthase inhibitors, such as ADMA, contribute to endothelial dysfunction [25], which is frequently encountered in heart failure [26]. Oechslin et al. have demonstrated that endothelial dysfunction is evident in adults with cyanotic congenital heart disease caused possibly by a reduced basal bioavailability of NO [27]. In summary, the unfavourable actions of ADMA are primarily the result of diminished NO availability, resulting in disturbed vasodilatation and anti-thrombotic, anti-inflammatory, and anti-apoptotic actions that overall might induce cardiac dysfunction [12]. Therefore, it is reasonable to assume that ADMA is involved in the dysfunction of various components of the cardiovascular system. That is why it probably better reflects the various pathophysiological changes involved in adults with congenital heart disease. In contrast, the BNP gene in cardiomyocytes is activated in response to increased myocardial wall stress due to volume- or pressure-overload states [28]. This results in the production of an intracellular precursor propeptide and after further processing in the release of the biologically inert aminoterminal fragment (NT-proBNP) and the biologically active BNP [28]. The half-life of NT-proBNP is longer than that of BNP, making it a better target for diagnostic blood testing. But in adults with congenital heart disease increased myocardial wall stress due to volume- or pressure-overload states is not always the mechanism of heart failure. For example, in patients with a single ventricle after the Fontan palliation the main mechanism of heart failure is a limitation of preload [29]. This could explain the limitations of NT-proBNP as a biomarker for heart failure in adults with congenital heart disease.

A limitation of this study is its cross-sectional design. To allow predictions about the prognostic value of elevated ADMA levels a longitudinal study is needed. This would be of great interest since previous studies suggest that NT-proBNP is not helpful in predicting clinical course of heart failure in ACHD [4]. Therefore, a long-term follow up study of the patients that participated in this study is already under way.
